# The Enzymology of 2-Hydroxyglutarate, 2-Hydroxyglutaramate and 2-Hydroxysuccinamate and Their Relationship to Oncometabolites

**DOI:** 10.3390/biology6020024

**Published:** 2017-03-30

**Authors:** Vivek A. Hariharan, Travis T. Denton, Sarah Paraszcszak, Kyle McEvoy, Thomas M. Jeitner, Boris F. Krasnikov, Arthur J. L. Cooper

**Affiliations:** 1 Department of Biochemistry and Molecular Biology, New York Medical College, Valhalla, NY 10590, USA; hariharanviv@gmail.com (V.A.H.); sparaszczak@umass.edu (S.P.); kyle_mcevoy@nymc.edu (K.M.); thomas_jeitner@nymc.edu (T.M.J.); boris_krasnikov@nymc.edu (B.F.K.); 2 Department of Pharmaceutical Sciences, Washington State University, College of Pharmacy, Spokane, WA 99210-1495, USA; travis.denton@wsu.edu

**Keywords:** ω-Amidase, asparagine transaminase, 2-hydroxyglutarate, 2-hydroxyglutaramate, 2-hydroxysuccinamate, glutamine synthetase, glutamine transaminases, 2-oxoglutaramate, 2-oxoglutarate

## Abstract

Many enzymes make “mistakes”. Consequently, repair enzymes have evolved to correct these mistakes. For example, lactate dehydrogenase (LDH) and mitochondrial malate dehydrogenase (mMDH) slowly catalyze the reduction of 2-oxoglutarate (2-OG) to the oncometabolite l-2-hydroxyglutarate (l-2-HG). l-2-HG dehydrogenase corrects this error by converting l-2-HG to 2-OG. LDH also catalyzes the reduction of the oxo group of 2-oxoglutaramate (2-OGM; transamination product of l-glutamine). We show here that human glutamine synthetase (GS) catalyzes the amidation of the terminal carboxyl of both the l- and d- isomers of 2-HG. The reaction of 2-OGM with LDH and the reaction of l-2-HG with GS generate l-2-hydroxyglutaramate (l-2-HGM). We also show that l-2-HGM is a substrate of human ω-amidase. The product (l-2-HG) can then be converted to 2-OG by l-2-HG dehydrogenase. Previous work showed that 2-oxosuccinamate (2-OSM; transamination product of l-asparagine) is an excellent substrate of LDH. Finally, we also show that human ω-amidase converts the product of this reaction (i.e., l-2-hydroxysuccinamate; l-2-HSM) to l-malate. Thus, ω-amidase may act together with hydroxyglutarate dehydrogenases to repair certain “mistakes” of GS and LDH. The present findings suggest that non-productive pathways for nitrogen metabolism occur in mammalian tissues in vivo. Perturbations of these pathways may contribute to symptoms associated with hydroxyglutaric acidurias and to tumor progression. Finally, methods for the synthesis of l-2-HGM and l-2-HSM are described that should be useful in determining the roles of ω-amidase/4- and 5-C compounds in photorespiration in plants.

## 1. Introduction

More than 90 years ago, Otto Warburg noticed that many cancer cells copiously metabolize glucose to lactate, even in the presence of adequate oxygen [1]. This process generates only two ATP molecules per molecule of glucose converted to lactate. This apparently wasteful process is still, however, adequate to meet most of the energy requirements of the cancer cells [2,3,4,5,6]. A major function of the mitochondria in many cancer cells is, therefore, to provide metabolic building blocks for rapidly dividing cells [6].

The unusual metabolism of many tumor cells is further exemplified by the production of oncometabolites (tumor transforming factors) such as l-2-hydoxyglutarate (l-2-HG; *S*-enantiomer), succinate and fumarate [7,8]. 2-Oxoglutarate (2-OG; α-ketoglutarate) is a poor substrate of mitochondrial malate dehydrogenase (mMDH) [9]. Nevertheless, mMDH is so abundant that this side reaction (Equation (1)) is not inconsequential. If there were no way to remove the l-2-HG produced, it would accumulate in tissues to potentially toxic levels. Thus, a repair enzyme (l-2-HG dehydrogenase (Equation (2))) has evolved to convert l-2-HG back to 2-OG.

2-Oxoglutarate (2-OG) + NADH + H^+^ ⇆ l-2-hydroxyglutarate (l-2-HG) + NAD^+^ (1)

l-2-HG + FAD → 2-OG + FADH_2_ (2)

l-2-Hydroxyglutaric aciduria is a rare inborn error of metabolism in which l-2-HG dehydrogenase is defective [9]. Children with l-2-hydroxyglutaric aciduria exhibit increased concentrations of l-2-HG in body fluids that are associated with progressive deterioration of central nervous system function, epilepsy, macrocephaly, leukoencephalopathy and a greatly increased risk of cerebral neoplasms [9,10,11,12]. l-2-HG inhibits 2-OG-utilizing enzymes, such as the Jumonji family of histone lysine demethylases [13]. Moreover, it has recently been shown that l-2-HG production is enhanced in hypoxic tissues [13], presumably as a result of increased cytosolic NADH and the ability of lactate dehydrogenase (LDH) to utilize 2-OG (albeit poorly) as a substrate [14]. Thus, l-2-HG may arise not only as a side reaction catalyzed by mMDH, but also as a side reaction catalyzed by LDH. The increase in l-2-HG during hypoxia appears to be sufficient to increase epigenetic methylation of histone repressive marks [13]. Moreover, the activity of mMDH and LDH toward 2-OG is stimulated several fold at acidic pH (relative to physiological pH) [14]. Acidic pH is known to stabilize hypoxia-inducible factor (HIF) [15]. This may be of significance in the acidic milieu of tumors with implications for HIF signaling [14].

l-2-HG has been proposed to serve an important role in the regulation of cellular redox homeostasis and cellular energy metabolism through its regulation of glycolysis and oxidative phosphorylation [16]. The concentration of l-2-HG was shown to be sensitive to metabolic perturbations in several mammalian cell lines in culture, and it was suggested that l-2-HG may play a critical role in the adaptive response of mammalian cells to hypoxia [16]. This may be especially relevant to tumor cells, which often grow in a hypoxic environment.

The d-enantiomer of 2-HG (d-2-HG; *R*-enantiomer) is normally produced in vivo by the action of hydroxy acid:oxo acid transhydrogenase (Equation (3)) [17]. In the brain and other organs, the succinic semialdehyde produced by this route is presumably converted to succinate which then enters the tricarboxylic acid (TCA) cycle via the GABA shunt. It has also been suggested that d-2-HG is generated in vivo as a metabolite of l-5-hydroxylysine (a component of collagen) [18] and possibly of aminolevulinate [19,20]. However, there is no known biological role for d-2-HG in normal tissues. Thus, this compound is salvaged by its conversion to 2-OG catalyzed by d-2-HG dehydrogenase (Equation (4)).

4-Hydroxybutyrate + 2-OG ⇆ succinic semialdehyde + d-2-hydroxyglutarate (d-2-HG) (3)

d-2-HG + FAD → 2-OG + FADH_2_ (4)

A rare inborn error of metabolism, in which d-2-HG dehydrogenase is defective, causes d-2-hydroxyglutaric aciduria (type I). The disease phenotype ranges from asymptomatic to severe neurological impairment, with symptoms ranging from early infantile-onset epileptic encephalopathy to hypotonia [21]. In another rare inborn error of metabolism (d-2-hydroxyglutaric aciduria type II), which is characterized by neurological problems, a mutation of isocitrate dehydrogenase 2 (IDH 2) causes a greatly enhanced ability of the enzyme to generate d-2-HG [22]. Mutations in either IDH1 or IDH 2 (found in several types of cancers, including gliomas [23,24,25,26]) result in not only loss of normal enzyme function (i.e., catalysis of the oxidative decarboxylation of isocitrate with concomitant production of NADH), but also to a pathological gain of a new enzyme function (i.e., reduction of 2-OG to the oncometabolite d-2-HG, while consuming NADH). Apparently, in patients and cancer cells harboring mutations in IDHs, the copious production of d-2-HG overwhelms the ability of d-2-HG dehydrogenase to convert d-2-HG back to 2-OG and d-2-HG may reach mM concentrations within tumors [25,26].

Despite the block in the TCA cycle, the mutations of IDH1/2 somehow convey a selective advantage to the tumor cells, likely by altering cellular epigenetics and/or blocking normal differentiation [23,24,25,26]. Many tumors make up for this loss of function by the up-regulation of key mitochondrial enzymes (e.g., glutaminase, glutamate dehydrogenase 1 and 2) to convert l-glutamine to 2-OG (“glutamine addiction”) [27,28]. Cancer cells harboring the IDH mutations, therefore, readily supply 5-C units in the form of 2-OG that can bypass the IDH 1/2 perturbation in the TCA cycle, thereby providing a means whereby d-2-HG can be produced without detriment to the cancer cells [23,24,25,26].

Recent work has suggested mechanisms whereby d-2-HG is oncogenic. For example, d-2-HG was shown to strongly inhibit (IC_50_ 25 μM) JMJD2A (a 2-OG-dependent enzyme of the Jumonji family of histone *N*^ε^-lysine demethylases) and, to a lesser extent, HIF prolyl hydroxylase (IC_50_ 5 mM) [29]. Decreased histone demethylation results in a block of cellular differentiation [30]. Koivunen et al. [31] showed that (*R*)-2-HG (i.e., d-2-HG), but not (*S*)-2-HG (i.e., l-2-HG), stimulates HIF prolyl hydroxylase 2 activity, leading to diminished HIF levels, which, in turn, enhances the proliferation and growth of human astrocytes in soft agar medium. Moreover, d-2-HG inhibits FTO (Fat Mass and Obesity-associated protein), a 2-OG-dependent dioxygenase that is located in the nucleus and is involved in the repair of single-stranded, alkylated RNA and DNA [32]. The main substrates of FTO likely are the 6-methyladenosine moieties in RNA [33]. Finally, production of d-2-HG by mutated IDH1/2 was shown to lead to the activation of mTOR by inhibiting KDM4A (lysine specific demethylase 4A; another 2-OG-dependent enzyme of the Jumonji family of *N*^ε^-lysine demethylases), an enzyme that specifically demethylates Lys 9 and 36 of histone H3 [34].

In yet another very rare inborn error of metabolism, D,l-2-hydroxyglutaric aciduria, both l-2-HG and d-2-HG enantiomers accumulate. Patients with combined D,l-2-hydroxyglutaric aciduria exhibit hypotonia, seizures and cerebral dysfunction [35,36]. The underlying molecular cause of this devastating disease is attributed to a mutation in the gene *SLC25A*, which codes for the mitochondrial citrate transport protein (mCTP) [35,36]. Recently, Jiang et al. [37] showed that mitochondrial metabolism in mCTP-deficient cancer cells is markedly altered. Thus, lactate, 2-d-HG and 2-l-HG are shown to accumulate, while as a consequence of inhibition of the pyruvate dehydrogenase complex, TCA cycle intermediates are depleted. In these cells, anaplerosis is stimulated by the activation of pyruvate carboxylase [37] and glucose is utilized to supply carbon for the mitochondrial production of 2-OG which, on entry to the cytosol, is used to power reductive carboxylation of 2-OG, by IDH1 (the cytosolic form of IDH), to citrate [37]. As stated by the authors: “the resulting citrate is cleaved to produce lipogenic acetyl-CoA, thereby completing a novel pathway of glucose-dependent reductive carboxylation. In CTP-deficient cells, IDH1 inhibition suppresses lipogenesis from either glucose or glutamine, implicating IDH1 as a required component of fatty acid synthesis in states of CTP deficiency” [37].

It is now evident that many metabolically important enzymes (e.g., mMDH and LDH mentioned above) are “promiscuous”. As a counter to this promiscuity, repair enzymes have evolved to prevent accumulation of wasteful/toxic metabolites generated from the promiscuity of these enzymes [38,39]. Here, we suggest additional possible cases of enzyme promiscuity and their possible enzymatic corrections.

More than 65 years ago, Meister showed that LDH has remarkably broad substrate specificity. For example, in addition to pyruvate, other 2-oxo acids such as glyoxylate, 3-hydroxypyruvate and 3-mercaptopyruvate were shown to be excellent substrates of crystalline beef heart LDH [40,41]. We have already alluded to the fact that LDH can catalyze the reduction of 2-OG to l-2-HG at a slow rate [40]. Moreover, Meister showed that LDH can also catalyze the slow reduction of 2-oxoglutaramate (2-OGM; α-ketoglutaramate; the 2-oxo acid formed by transamination of l-glutamine) [42]. Meister also showed that 2-oxosuccinamate (2-OSM; α-ketosuccinamate; the 2-oxo acid formed by transamination of l-asparagine) is an excellent substrate of LDH [42]. The products of these two reactions are presumably l-2-hydroxyglutaramate (l-2-HGM) and l-2-hydroxysuccinamate (l-2-HSM), respectively. Glutamine synthetase (GS; glutamate-ammonia ligase) is also a promiscuous enzyme. For example, in addition to utilizing l-glutamate as a substrate, the sheep brain enzyme also effectively utilizes d-glutamate [43]. Moreover, chicken liver GS utilizes not only d-glutamate as a substrate but also l-2-HG [44]. The product of the reaction with l-2-HG is presumably l-2-HGM. In the current work, we present evidence that human GS also utilizes l-2-HG as a substrate, to a limited extent.

ω-Amidase (systematic name: ω-amidodicarboxylate amidohydrolase) (synonym: Nit2, nitrilase-like protein 2) normally catalyzes the hydrolysis of 2-OGM to 2-OG (Equation (5)) and 2-OSM to oxaloacetate (Equation (6)), respectively (see also the Discussion). 2-OG and oxaloacetate can then enter the TCA cycle as an energy source.

2-Oxoglutaramate (2-OGM) + H_2_O → 2-oxoglutarate (2-OG) + NH_3_ (5)

2-Oxosuccinamate (2-OSM) + H_2_O → oxaloacetate + NH_3_ (6)

Here, we show that the corresponding l-2-hydroxy acid analogues of l-glutamine and l-asparagine (i.e., l-2-HGM and l-2-HSM, respectively) are also substrates of human ω-amidase. The corresponding reactions (water as attacking nucleophile) are shown in Equations (7) and (8), respectively.

l-2-Hydroxyglutaramate (l-2-HGM) + H_2_O → l-2-hydroxyglutarate + NH_3_ (7)

l-2-Hydroxysuccinamate (l-2-HSM) + H_2_O → l-malate + NH_3_ (8)

In the case of l-2-HSM, the compound is converted by ω-amidase to l-malate, which can then enter the TCA cycle. In the case of l-2-HGM, the compound is converted to l-2-HG, which will then be converted to 2-OG by l-2-HG dehydrogenase (Equation (2)), which can then enter the TCA cycle. Thus, in addition to catalyzing metabolically important reactions (hydrolysis of 2-OGM and 2-OSM to 2-OG and oxaloacetate, respectively) ω-amidase may also be regarded as a repair enzyme for salvaging l-2-HSM (as l-malate), and, working in conjunction with l-2-HG dehydrogenase, for salvaging l-2-HGM (as 2-OG).

## 2. Materials and Methods

### 2.1. Reagents

All reagents were of the highest quality available. Bovine serum albumin (fraction V), dithiothreitol (DTT), NAD^+^, l-glutamine, l-asparagine, l-malate, sodium 2-OG, potassium phosphate, hydroxylamine hydrochloride (stock solutions neutralized with NaOH before use), tris hydrochloride, glycine, sodium hydroxide, ferric chloride, lithium lactate, trichloroacetic acid, glycylglycine and protease inhibitor cocktail were obtained from Sigma Aldrich Chemical Company (St. Louis, MO, USA). Succinamic acid was obtained from Aldrich Chemical Company (Milwaukee, WI, USA). 2,4-Dinitrophenylhydrazine was obtained from Eastman Kodak (Rochester, NY, USA). Stock solutions of 2-OGM, in which the pH was adjusted to around 6.0 with NaOH, were prepared by the method of Krasnikov et al. [45].

### 2.2. Enzymes

Human ω-amidase/Nit2 (0.29 μg/μL) was purchased from Origene Technologies, Inc., Rockville, MD. The preparation exhibits a single band on PAGE and is enzymatically active (data not shown). Pig heart mMDH (3.2 mg protein/mL in 50% glycerol, 50 mM potassium phosphate, pH 7.5) was purchased from Sigma-Aldrich, St. Louis, Mo. Human GS was expressed and purified by the method of Listrom et al. [46] except that during the purification procedure HA Ultrogel^®^ (Pall Life Sciences; Pall Biosepra, Saint Cristophe, France) was used in place of hydroxylapatite [47]. The specific activity of purified recombinant human GS (ammonia and glutamate as substrates) was 17.9 U/mg [47,48].

### 2.3. Preparation of Rat Liver Cytosol

The procedure used for the isolation of cytosol from the liver of a single adult male rat was as described by Krasnikov et al. [49].

### 2.4. Enzyme Assays

#### 2.4.1. ω-Amidase

As noted above, ω-amidase catalyzes the hydrolysis of 2-OGM to 2-OG (Equation (5)) and the hydrolysis of 2-OSM to oxaloacetate (Equation (6)). The hydrolysis of 2-OGM to 2-OG was measured by the procedure of Krasnikov et al. [45] as follows: The reaction mixture contained 3 mM 2-OGM, 5 mM DTT, 100 mM tris-HCl buffer (pH 8.0) and enzyme in a final volume of 50 μL. After incubation at 37 °C, the reaction was quenched by addition of 20 μL of 5 mM 2, 4-dinitrophenylhydrazine in 2 M HCl. After a further incubation at 37 °C for 5 min, 130 μL of 1 M NaOH was added and the absorbance was read at 430 nm after 5 min (ε 2-OG 2, 4-dintrophenylhydrazone ~16,000 M^-1^·cm^-1^). It should be noted that although the open-chain form of 2-OGM contains a reactive carbonyl, at physiological pH values and in the presence of the acidic reagent, most of the compound is cyclized to a lactam [45,50,51] that does not react with 2, 4-dinitrophenylhydrazine.

In addition to catalyzing the hydrolysis of 2-OGM and 2-OSM, rat liver ω-amidase also catalyzes the hydroxaminolysis of succinamate [42,45,50,51] (Equation (9)). The resulting hydroxamate is readily quantitated as a brown complex in the presence of highly acidic ferric chloride [45]. In the present work, this procedure was adapted to measure the hydroxaminolysis of l-2-HGM (Equation (10)).

Succinamate + NH_2_OH → 3-succinylhydroxamate + NH_3_ (9)

l-2-Hydroxyglutaramate (l-2-HGM) + NH_2_OH → 2-hydroxy-4-glutamylhydroxamate + NH_3_ (10)

The reaction mixture (50 μL) contained 50 mM l-2-HGM, 100 mM potassium phosphate buffer (pH 7.4), 1 mM DTT, 100 mM hydroxylamine, enzyme and, where indicated, glycylglycine (an inhibitor of ω-amidase; see below). After incubation at 37 °C for 1 hour, the reaction was terminated by addition of 0.15 mL of FeCl_3_ reagent (0.37 M FeCl_3_, 0.67 M HCl, 0.2 M trichloroacetic acid [45]). Precipitated protein (when present) was removed by centrifugation (10 min, 10,000 *g*) and the absorbance of the 0.18 mL of the supernatant was determined at 535 nm. The extinction coefficient at 535 nm of the ferric complex of the succinylhydroxamate was previously calculated to be 920·M^-1^ cm^-1^ [45]. The blank lacked either hydroxylamine or enzyme. In the present work, the extinction coefficient of the ferric complex of l-2-HG hydroxamate at 535 nm is also assumed to be 920 M^-1^·cm^-1^.

The hydroxamate assay could not be used to determine whether l-2-HSM is a substrate of human ω-amidase (see the results). Therefore, we used an alternative assay to determine whether l-2-HSM is a substrate of human ω-amidase. The reaction mixture (50 μL) contained 50 mM l-2-HSM, 100 mM potassium phosphate buffer, 1 mM DTT and 146 ng of enzyme. After incubation at 37 °C for 2 h, the reaction was quenched by addition of 140 μL of glycine-NaOH buffer (pH 10.0) followed by 5 μL of NAD^+^ and 5 μL of MDH solution. The increase in absorbance at 340 nm (due to formation of NADH; ε 6.22 × 10^3^ M^-1^·cm^-1^) was measured relative to that of a blank that lacked enzyme and compared to values on a standard curve generated with various concentrations of l-malate in a reaction mixture lacking ω-amidase.

#### 2.4.2. Glutamine Synthetase (GS)

GS activity was determined by the end-point γ-glutamylhydroxamate assay [43] as modified by Jeitner and Cooper [47] and Jeitner et al. [48] and is as follows: The reaction mixture (60 μL) contained 100 mM tris-HCl, 10 mM MgCl_2_, 10 mM ATP, 100 mM hydroxylamine, 2 mM DTT, 5 μL (9 μg) GS and varying amounts of l-glutamate. The reaction was initiated by the addition of enzyme solution. After incubation at 37 °C for various time intervals, the reaction was quenched by the addition of 180 μL of a solution containing 0.37 M ferric chloride, 0.67 M HCl and 0.2 M trichloroacetic acid [43]. After centrifugation at 10,000 *g* for 5 min at 4 °C, to remove precipitated protein, the absorbance of 200 μL of the supernatant fraction at 535 nm was recorded. The amount of γ-glutamyl hydroxamate formed from l-glutamate under these conditions was calculated using 850 M^-1^·cm^-1^ as the extinction coefficient [52]. In some experiments, l-glutamate was replaced by either l-2-HG or d-2-HG. The extinction coefficient at 535 nm of the resulting ferric chloride hydroxamate complex is assumed to be 850 M^-1^·cm^-1^.

### 2.5. Preparation of l-2-Hydroxyglutaramic Acid (l-2-HGM; S-5-Amino-2-Hydroxy-5-Oxopentanoic Acid)

**Scheme 1:**
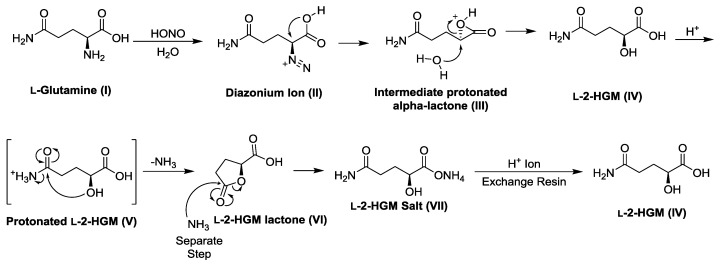
Protocol for the synthesis of l-2-hydroxyglutaramic acid (l-2-HGM).

The hydroxy acids used in this study are to be considered stereochemically pure, in accordance with previously published, accepted considerations. The reaction mechanism for the preparation of l-2-HGM from l-glutamine, as shown below in Scheme 1, is an example. After diazotization of the amino nitrogen of l-glutamine, the carboxylic acid moiety of carbon-1 attacks, in an S_N_2 fashion, to invert the stereochemistry at the 2-carbon, while creating the intermediate, protonated α-lactone (Intermediate III), which subsequently reacts with a solvent molecule (water) in a second, S_N_2-mediated substitution reaction to form l-2-HGM with overall retention of stereochemistry. Due to the reaction conditions, and to the stability of the product formed, the l-2HGM undergoes an acid-catalyzed cyclization to afford the γ-lactone VI, which is subsequently aminated with aqueous ammonia to afford the salt VII, which is ultimately protonated with an ion exchange resin to afford l-2-HGM, after removal of the solvent and trituration with ethanol. (The use of an ion exchange resin affords protons to remove any excess ammonia and to neutralize the l-2-HGM salt, while not providing a large excess of solubilized protons to catalyze the cyclization for a second time.)

The experimental protocol for the synthesis of l-2-HGM is as follows: To a solution of l-glutamine (4.10 g, 28.1 mmol) in a 100 mL solution of acetic acid in water (20:80/v:v) at 0 °C was added, dropwise, a solution of sodium nitrite (1.94 g, 28.1 mmol) in water (25 mL) over a 1 h period. Once the addition was complete, the solution was stirred at ambient temperature for 18 h. The volatiles were removed by rotary evaporation (35 °C; water bath) and transferred, with the aid of ca. 5 mL of water, via a glass pipette, to a stirring solution of ammonia in water (ammonium hydroxide, ca. 18 M, 36.5 mL, ca. 657 mmol) and the resultant solution was stirred at room temperature for 24 h. The solvent and excess ammonia were removed via rotary evaporation, the residue was dissolved in ca. 10 mL of water and applied to a column containing well washed Dowex 50W-X8 ion exchange resin (ca. 50 mL, ca. 100 mmol H^+^) and eluted with water. The eluent with a pH below ca. 4 was collected and the volatiles were removed via rotary evaporation to afford the crude material (3.77 g, 91.3% crude yield) as a sticky white solid. The residue was triturated with 200 proof ethanol, the solid material was collected by filtration and the residual solvent was removed *in vacuo* to afford l-2-HGM (2.34 g, 54.2% yield) as a white solid: ^1^H NMR (500 MHz, D_2_O) δ = 4.14 (dd, J = 4.6, 7.7 Hz, 1 H), 2.31–2.18 (m, 2 H), 1.96 (dddd, J = 4.6, 7.2, 8.7, 14.2 Hz, 1 H), 1.85–1.76 (m, 1 H); ^13^C NMR (100 MHz, D_2_O (0.5% t-BuOH)) δ = 179.6, 178.5, 70.45, 31.66, 30.39. HRMS (ESI) *m*/*z* calcd for C_5_H_10_NO_4_ [M + H]^+^ 148.0610, found 148.0609. The ^1^H NMR and ^13^C NMR spectra are shown in supplemental Figures S1A and S1B, respectively.

### 2.6. Preparation of l-2-Hydroxysuccinamic Acid (l-2-HSM; S-4-Amino-2-Hydroxy-4-Oxobutanoic Acid)

**Scheme 2:**
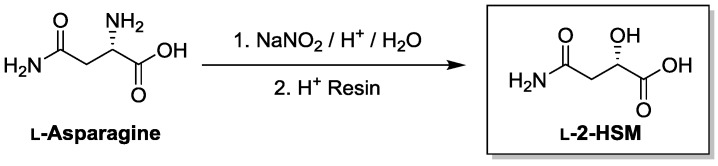
Protocol for the synthesis of l-2-hydroxysuccinamic acid (l-2-HSM).

In the case of the preparation of l-2-HSM, the second step of the procedure as described above for the preparation of l-2-HGM is not necessary, as the product l-2-HSM does not cyclize to form any detectable β-lactone. The experimental procedure is as follows: To a slurry of l-asparagine (7.80 g, 59.0 mmol) in a solution of acetic acid in water (20:80/v:v, 100 mL) at 0 °C was added a solution of sodium nitrite (4.89 g, 70.8 mmol) in water (20 mL) in one portion. The resultant mixture was stirred for 18 h. The red mixture was applied to a column containing well washed Dowex 50W-X8 ion exchange resin (200 mL, ca. 120 mmol H^+^) and eluted with water. The eluent was collected and the volatiles were removed via rotary evaporation (50 °C; water bath) to afford the crude material as a red solid. The residue was triturated with 200 proof ethanol, the solid material was collected by filtration and the residual solvent was removed *in vacuo* to afford l-2-HSM (4.97 g, 63.3% yield) as an off white solid: ^1^H NMR (500 MHz, D_2_O) δ = 4.41 (dd, J = 4.4, 7.9 Hz, 2 H), 2.63 (dd, J = 4.4, 15.4 Hz, 2 H), 2.52 (dd, J = 7.7, 15.3 Hz, 2 H); ^13^C NMR (100 MHz, D_2_O (0.5% t-BuOH)) δ = 177.7, 176.2, 68.2, 40.35. HRMS (ESI) *m*/*z* calcd for C_4_H_8_NO_4_ [M + H]^+^ 134.0718, found 134.0795. The synthesis of l-2-HSM is summarized in Scheme 2. The ^1^H NMR and ^13^C NMR spectra are shown in supplemental Figures S2A, and S2B, respectively.

2-HSM has been prepared previously by reduction of 2-OSM with 0.2 M sodium borohydride [53,54]. Thus, the product was presumably a mixture of d- and l-enantiomers. Moreover, 2-OSM used in this procedure was generated enzymatically. The present procedure is an improvement over the previous method because it does not involve an enzymatic step (and therefore can be used in scaled up procedures that are not feasible by enzymatic procedures). Additionally, the product generated by the present procedure is to be considered of the l-configuration.

### 2.7. Preparation of the l- and d-Enantiomers of 2-Hydroxyglutaric Acid

d- and l-2-hydroxyglutaric acids were prepared from (*R*)-(+)-5-oxo-tetrahydrofurancarboxylic and (*S*)-(-)-5-oxo-tetrahydrofurancarboxylic acids (Sigma-Aldrich, St. Louis, MO, USA), respectively (Supplementary Materials).

### 2.8. Spectrophotometry

All spectrophotometric measurements were carried out with a SpectraMax M5 96-well plate spectrophotometer (Molecular Devices, Sunnyvale, CA, USA).

### 2.9. Statistics

Data are reported as the mean ± S.D. All enzyme assays were carried out in at least triplicate. The unpaired t-test was used to determine significance values between means. A *p* value ≤ 0.05 is considered significant.

## 3. Results

### 3.1. Human ω-Amidase Utilizes l-2-Hydroxyglutaramate (l-2-HGM) and l-2-Hydroxysuccinamate (l-2-HSM) as Substrates

As noted above, rat liver ω-amidase catalyzes the hydrolysis of 2-OGM and 2-OSM to 2-OG and oxaloacetate, respectively [42,45,55,56,57,58,59] (Equations (5) and (6)). Moreover, rat liver ω-amidase also catalyzes the hydroxaminolysis of succinamate and glutaramate [50,51]. Based on these findings, we developed convenient assays to measure 2-OG formation from 2-OGM and the hydroxaminolysis of succinamate [45]. These assays were employed in the present work to show that human ω-amidase, similar to the rat liver enzyme, catalyzes the hydrolysis of 2-OGM and the hydroxaminolysis of succinamate (Table 1). It was previously shown that neither l-glutamine nor l-asparagine is a detectable substrate of semi-purified rat liver ω-amidase [57]. Presumably, the charge on the α-amino group of l-glutamine/l-asparagine prevents effective binding of this moiety to the region of the active site that normally binds the oxygen of the of the 2-oxo group of the substrates 2-OGM/2-OSM. Thus, it was of interest to determine whether l-2-HGM (in which the 2-oxo group of 2-OGM is replaced with an uncharged 2-hydroxy group) is a substrate of human ω-amidase (hydroxaminolysis procedure). Table 1 shows that l-2-HGM is indeed a substrate of human ω-amidase.

Based on the findings shown in Table 1, that succinamate and l-2-HGM are substrates of human ω-amidase (hydroxaminolysis reaction), we fully expected this enzyme to catalyze the hydroxaminolysis of l-2-HSM. However, when the enzyme was incubated with l-2-HSM and hydroxylamine in the presence of human ω-amidase, no product that absorbs at 535 nm upon addition of acidic FeCl_3_ reagent could be detected (data not shown). As a consequence, we used another procedure to determine whether l-2-HSM is a substrate of human ω-amidase. l-Malate was detected as a product with mMDH (Table 1) confirming that l-2-HSM is a substrate of human ω-amidase (Equation (8)).

We suspect that the hydroxamate formed with l-2-HSM cyclizes to a compound (5-hydroxy-1,2-oxazinane-3,6-dione) that does not form a colored complex with the acidic FeCl_3_ reagent. It was previously shown that l-γ-glutamyl hydroxamate is a substrate of l-amino acid oxidase [58]. The product was not characterized, but, as noted for 2-OGM [50,51], it is expected to cyclize to a lactam (in this case 1,2-dihydroxy-5-oxoproline). This compound is also expected to arise from the hydroxaminolysis of 2-OGM. When we incubated 5 µL of rat liver cytosol with 3 mM 2-OGM in the presence of 100 mM hydroxylamine and tris buffer (pH 8.0) for one h at 37 °C no product that absorbs at 535 nm could be detected upon addition of the acidic FeCl_3_ reagent (data not shown). Under these conditions, products absorbing at 535 nm were readily detected when 2-OGM was replaced by either succinamate or l-2-HGM (Table 2). Thus, cyclized hydroxamates (5-hydroxy-1,2-oxazinane-3,6-dione and 1,2-dihydroxy-5-oxoproline) do not form a complex with FeCl_3_ that absorbs at 535 nm.

### 3.2. Rat Liver Cytosol Catalyzes the Hydroxaminolysis of l-2-Hydroxyglutaramate (l-2-HGM)

The ability of a rat liver cytosolic preparation to catalyze the hydroxaminolysis of l-2-HGM was tested. As a positive control, we first verified that this preparation catalyzes the hydroxaminolysis of succinamate, an alternative ω-amidase substrate (Table 2). Glycylglycine is a competitive inhibitor of rat liver ω-amidase with a *K*_i_ of around 3 mM [60]. This compound was shown to inhibit the hydroxaminolysis of succinamate (Table 2), consistent with the presence of ω-amidase in the rat liver cytosol. l-2-HSM was also found to inhibit the hydroxaminolysis of succinamate (Table 2). This finding is consistent with the hypothesis that l-2-HSM acts as a competitive inhibitor of the ω-amidase-catalyzed hydroxaminolysis of succinamate because it is an alternative substrate.

As expected, based on the present findings that highly purified human ω-amidase catalyzes a hydroxaminolysis reaction with l-2-HGM (Table 1), the rat liver cytosol preparation was also shown to catalyze a hydroxaminolysis reaction with l-2-HGM (Table 2). Evidence that this reaction is catalyzed by ω-amidase is the finding that, as with the hydroxaminolysis of succinamate, the hydroxaminolysis reaction with l-2-HGM is inhibited by both glycylglycine and l-2-HSM (Table 2). Based on our findings with the human enzyme, ω-amidase in the rat liver cytosol is expected to catalyze the conversion of l-2-HSM to malate. However, we could not measure this reaction due to enzymes in the cytosolic preparation that interfered with the mMDH used to quantitate the product (data not shown). Nevertheless, the possibility that l-2-HSM binds at the active site of ω-amidase in the rat liver homogenate is indicated by its inhibition of (1) the hydroxaminolysis reaction with both succinamate and l-2-HGM (Table 2) and (2) the hydrolysis of 2-OGM (Table 3).

It is possible that glycylglycine, succinamate and/or l-2-HSM may interact with enzymes other than ω-amidase in the rat liver homogenate to generate products that indirectly inhibit the ω-amidase reaction. Although we cannot fully rule out this possibility, the data in Table 2 and Table 3 are fully consistent with patterns of inhibition noted with purified ω-amidase preparations.

In summary, l-2-HGM was shown directly to be a substrate of human ω-amidase and of an enzyme in rat liver cytosol (presumably ω-amidase). In addition, highly purified human ω-amidase was shown to catalyze the hydrolysis of l-2-HSM to l-malate (Table 1). Moreover, l-2-HSM was shown to inhibit the hydroxaminolysis of succinamate in rat liver cytosol (Table 2). Finally, strong but indirect evidence suggests that l-2-HSM binds to the rat liver enzyme in the liver homogenate most likely as an alternative substrate to 2-OGM/succinamate (Table 3).

### 3.3. l- and d-2-Hydroxyglutarate Are Weak Substrates of Human Glutamine Synthetase (GS)

**Figure 1:**
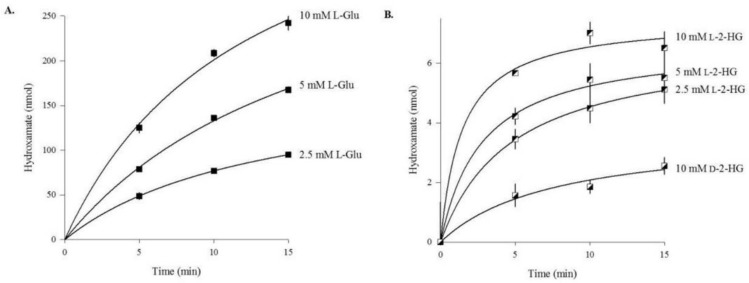
Substrate behavior of human glutamine synthetase (GS) toward 2-hydroxyglutarate (2-HG) enantiomers. Progress curves are shown for the formation of hydroxamate from either l-glutamate (l-Glu) (Panel **A**) or from l-2-HG and d-2-HG (Panel **B**). The means and SE of three independent observations are shown. Note that the scale on the Y axis in panel B is less than that of panel A to accommodate the low rate of GS-catalyzed hydroxamate formation from the 2-HG enantiomers.

As noted in the Introduction, unlike most enzymes that utilize amino acids as substrates, where there is an absolute requirement for either the d- or l-enantiomer, sheep brain GS can effectively utilize both the l- and d- enantiomers of glutamate as substrates [43]. As also mentioned, the chicken liver enzyme has some activity with l-2-HG [44]. Thus, it was of considerable interest to determine whether both the l- and d-2-HG enantiomers are substrates of human GS (Figure 1).

The progress curves for l-glutamate as a substrate (Figure 1A) of human GS are similar to those we reported previously and are consistent with slowing of the reaction with time due to depletion of substrate [47]. Although no rigorous kinetic analyses are shown, the data clearly show that both enantiomers of 2-HG are substrates of human GS (Figure 1B). Under the conditions of the assay, the l-enantiomer is a better substrate than is the d-enantiomer. However, the rate of hydroxamate formation with the two 2-HG enantiomers (Figure 1B) is much slower than that observed with l-glutamate (Figure 1A). Interestingly, the rate of the reaction with d- and l-2-HG was found to slow considerably with time. This finding is reminiscent of our previous findings with the two diastereoisomers of l-4-fluoroglutamate (i.e., 2*S*, 4*R*-4-fluoroglutamate and 2*S*, 4*S*-4-fluoroglutamate) which are detectable substrates of human GS, but share the distinguishing feature in which the rate of the enzyme-catalyzed reaction rapidly decreases with time [48]. The mechanism(s) resulting in the apparent inactivation of human GS by the two enantiomers of 2-HG and by the two diasteroisomers of l-4-fluoroglutamate require(s) further study.

## 4. Discussion

Mammalian tissues contain at least two glutamine transaminases that catalyze the transamination of l-glutamine to 2-OGM (Equation (11)). When coupled with the ω-amidase reaction (Equation (5)) the net reaction (Equation (12)) constitutes the glutaminase II pathway [55,56,57,58,59].

l-Glutamine + 2-oxo acid ⇆ 2-oxoglutaramate (2-OGM) + l-amino acid (11)

2-OGM + H_2_O → 2-OG + NH_3_

Net: l-Glutamine + 2-oxo acid + H_2_O → 2-OG + l-amino acid + NH_3_ (12)

Mammalian tissues also catalyze the transamination of l-asparagine to 2-OSM (Equation (13)). 2-OSM is then hydrolyzed to oxaloacetate and ammonia by ω-amidase (Equation (6)). The net reaction (Equation (14)) is known as the asparaginase II pathway [56,60].

l-Asparagine + 2-oxo acid ⇆ 2-oxosuccinamate (2-OSM) + l-amino acid (13)

2-OSM + H_2_O → oxaloacetate + NH_3_

Net: l-Asparagine + 2-oxo acid + H_2_O → oxaloacetate + l-amino acid + NH_3_ (14)

As a result of the cyclization of 2-OGM and the high activity of ω-amidase, the glutamine transaminase reaction is largely irreversible in mammalian tissues. Moreover, the glutamine transaminases have wide 2-oxo acid substrate specificity. Thus, one of the biological roles of the mammalian glutamine transaminases may be to salvage 2-oxo acids produced through non-specific transamination reactions (“mistakes”) catalyzed by other aminotransferases [61]. The glutaminase II pathway (i.e., glutamine transaminases plus ω-amidase) also closes the methionine salvage pathway by transaminating 2-oxo-(4-methylthio)butanoate (α-keto-γ-methiolbutyrate) to l-methionine [61]. Additionally, the kidney type glutamine transaminase catalyzes transamination of a number of sulfur-containing amino acids to compounds that cyclize to products, several of which may be neuroactive [62].

The asparaginase II pathway is important in photorespiratory nitrogen metabolism in plants [53,54,63]. However, little is known about the metabolic importance of the asparaginase II pathway in mammals. One study suggests that the asparaginase reaction (Equation (15)) is the major route for the metabolism of asparagine in rat liver cytosol, whereas the asparaginase II pathway (Equation (13)) is the major route in rat liver mitochondria [64].

l-Asparagine + H_2_O → l-aspartate + NH_3_ (15)

Transamination of asparagine to 2-OSM presents a potential problem. 2-OSM is highly reactive, forming a dimer in vitro [65,66]. The dimer can undergo ring closures, dehydration, aromatization, deamidation and decarboxylation to a plethora of heterocyclic compounds [65,66]. Even if the concentration of 2-OSM in vivo is too dilute for an effective bimolecular reaction, it is possible that the compound could react with other α-keto acids or other reactive biomolecules. One of the roles of ω-amidase may, therefore, be to prevent the accumulation of 2-OSM and condensation of 2-OSM with another molecule of 2-OSM (or other reactive compound). Indeed, ω-amidase has a noticeably low *K*_m_ toward 2-OSM (~3 μM for the mouse enzyme at pH 7.2) [59].

We suggest that another way to prevent the accumulation of 2-OSM is to reduce 2-OSM to l-2-HSM. As noted above, 2-OSM is a good substrate of LDH [42]. In the present work, we show that l-2-HSM is a substrate of ω-amidase. Thus, 2-OSM may be converted to oxaloacetate via the action of ω-amidase, or it may first be reduced to l-2-HSM by LDH, followed by ω-amidase-catalyzed hydrolysis of l-2-HSM to l-malate. Both products (i.e., oxaloacetate and malate) are useful TCA cycle metabolites. In this regard, it is interesting to note that l-2-HSM has been shown to be a substrate of plant ω-amidases [54,67]. Given that mammalian ω-amidases utilize both 4- and 5-C substrates, it is highly likely that l-2-HGM will also be a substrate of these plant ω-amidases. This hypothesis is supported by the finding that human ω-amidase utilizes l-2-HGM as a substrate (Table 1).

It is of interest that, as noted above, an avian GS utilizes l-2-HG as a substrate [44]. In the present work, we show that both the l- and d- enantiomers of 2-HG are also substrates of human GS (hydroxamate assay), but the relative rate with the l- and d- enantiomers of 2-HG is relatively slow (initial rates are a few percent relative to that exhibited with l-glutamate; Figure 1) compared with that exhibited by the avian enzyme toward l-2-HG (~41% relative to that exhibited with l-glutamate (44)). Hydroxylamine and ammonia are nearly kinetically indistinguishable as substrates of ovine brain GS [43]. This is also very likely to be the case with the human enzyme. Thus, the present work strongly suggests that human GS can catalyze the formation of d- and l-2-HGM from d- and l-2-HG, respectively, albeit at a considerably slower rate than that observed with l-glutamate. The difference in l-2-HG utilization by the human and avian glutamine synthetases may be due to a difference in the active sites. Binding of l-glutamate to the human enzyme is mediated by Arg^340^, His^253^ and Arg^299^ [68]. Avian glutamine synthetase, however, has a glycine substitution at position 340. In human glutamine synthetase, the guanidino group of Arg^340^ interacts with the γ-carboxyl group of l-glutamate thereby stabilizing this amino acid within the substrate binding site. The substitution of Gly for Arg at this position removes a crucial contact site for l-glutamate and, likely, for l-2-HG, in the avian enzyme. In support of this conclusion, the *K*_m_ values for l-glutamate are 1.67 mM and 6.20 mM for human and avian glutamine synthetase, respectively [44,47]. The Arg^340^ to Gly^340^ substitution may also facilitate the catalysis of l-2-HG to l-2-HGM by avian glutamine synthetase. This substitution removes a bulky, charged side chain that may facilitate either access of ammonia to the phosphorylated intermediate of glutamate or the egress of l-2-HGM from the active site. However, a detailed comparative analysis of the topology of the active sites of the human and avian enzymes must await further study.

We also noted above that 2-OGM is a weak substrate of LDH [42]. Thus, l-2-HGM may arise from side reactions in mammalian tissues through at least two enzymatic reactions (i.e., via a side reaction of GS with l-2-HG or by reduction of 2-OGM by LDH). In the current work, we show that l-2-HGM is a substrate of human ω-amidase. Strong enzymatic activity toward l-2-HGM was also noted in rat liver homogenates, most likely due, in large part, to endogenous ω-amidase. Thus, we propose that mammalian ω-amidase not only converts 2-OGM directly to metabolically useful 2-OG, but also acts in conjunction with l-2-HG dehydrogenase to metabolize l-2-HGM to 2-OG.

Interestingly, it has recently been found that enzymes in the serine biosynthetic pathway in *Saccharomyces cerevisiae* can generate d-2-HG [69]. This d-2-HG can then be converted to 2-OG by an FAd-dependent transhydrogenase that uses pyruvate as a hydrogen acceptor. The d-2-HG in the cytosol is coupled to the shuttling of reducing equivalents from cytosolic NADH to the mitochondrion via d-lactate dehydrogenase. Thus, in this case, production of d-2-HG is part of a useful biochemical mechanism.

## 5. Conclusions

**Figure 2:**
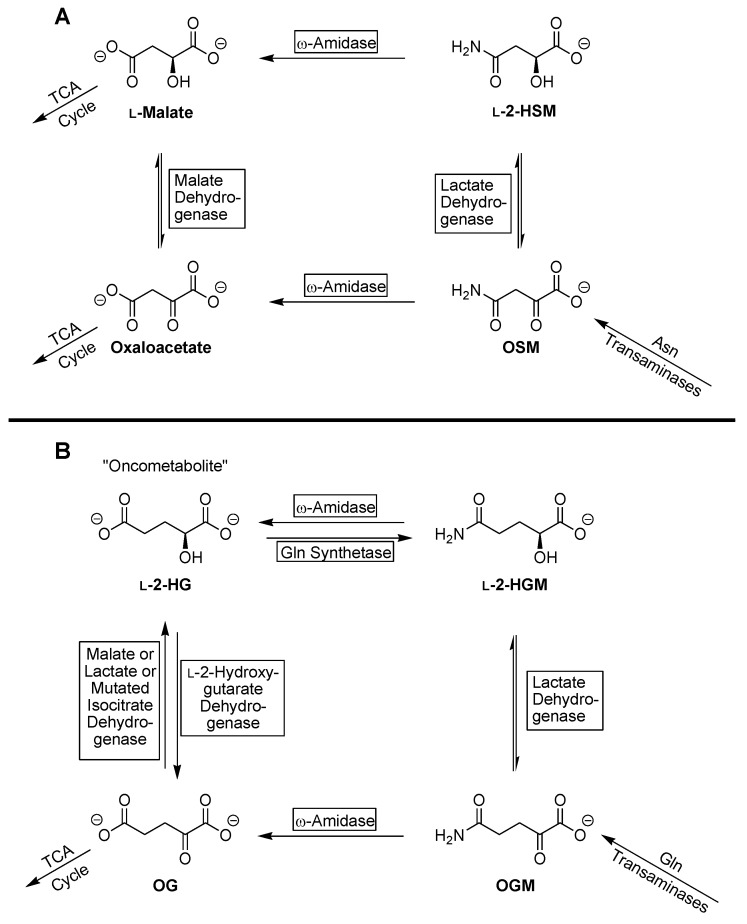
Metabolically important reactions and side reactions involved in the metabolism of 2-oxosuccinamate (2-OSM) (**A**) and 2-oxoglutaramate (2-OGM) (**B**). The asparaginase II pathway (bottom of panel A) and the glutaminase II pathway (bottom of panel B) may be regarded as major metabolic pathways for l-asparagine and l-glutamine, respectively. On the other hand, excessive formation of l-2-HSM and l-2-HGM as side reactions may be regarded as metabolic mistakes, so that repair pathways for their conversion to metabolically useful l-malate/oxaloacetate (Panel A) and 2-OG (Panel B) are required. Not shown is the formation of d-2-HG by mutant IDHs and the possible utilization of this compound as a substrate of GS (Gln Synthetase). For clarity, some cofactors and co-substrates have been omitted from the diagram.

The present work has uncovered some interesting enzyme-catalyzed side reactions (and also describes some additional side reactions “buried” in the literature). However, no detailed kinetic analyses were performed in the present work. Nevertheless, because the enzyme side reactions investigated here/described in the literature are catalyzed by enzymes of inherently very high activity (e.g., LDH, GS and ω-amidase), we believe that the side reactions may indeed occur in vivo, albeit at a low rate. We suggest that the side reactions uncovered in the present work (summarized in Figure 2) should act as a framework for future studies and be taken into account when studying the metabolic profiles of tumor cells. Although we have assumed that formation of l-2-HSM and l-2-HGM are metabolic mistakes (Figure 2, legend), and that repair enzymes are necessary to correct these mistakes, we cannot rule out the possibility that these compounds may have some unknown, but useful, function. We also cannot rule out the possibility that they may contribute to the oncogenic properties of small 4-C and 5-C metabolites. Thus, in summary, the present work may serve as a basis for the discovery of biologically important processes that hitherto were assumed to have no biological relevance. Finally, we provide organic synthesis procedures for the preparation of l-2-HGM and l-2-HSM, which should be useful for further metabolic studies of these compounds in plants, mammals and other organisms.

## Figures and Tables

**Table 1:** Substrates of human Nit 2/ω-amidase.

Substrate	Enzyme Added (ng)	Specific Activity μmol/min/mg ^a^
2-Oxoglutaramate (2-OGM) ^b^	72.8	3.0 ± 0.9 (3)
Succinamate ^c^	146	3.4 ± 0.3 (3)
l-2-Hydroxyglutaramate (l-2-HGM) ^c^	291	1.4 ± 0.1 (7)
l-2-Hydroxysuccinamate (l-2-HSM) ^d^	146	0.51 ± 0.18 (5)

^a^ n, the number of replicates is shown in parenthesis. After incubation for 1 to 2 hours, product formation was determined from which the specific activity was calculated. In a separate experiment, it was shown that the reactions listed are linear over this time period. ^b^ The reaction mixture (50 μL) contained 100 mM tris buffer (pH 8.0), 12.6 mM 2-OGM, 1 mM DTT and enzyme (as indicated). After incubation at 37 °C, 2-OG formation was measured. ^c^ The reaction mixture (50 μL) contained 100 mM potassium phosphate buffer (pH 7.4), 50 mM substrate, 100 mM hydroxylamine, 1 mM DTT and enzyme (as indicated). After incubation at 37 °C hydroxamate formation was measured with FeCl_3_ reagent. ^d^ The reaction mixture (50 μL) contained 100 mM potassium phosphate buffer (pH 7.4), 50 mM substrate, 1 mM DTT and enzyme (as indicated). After incubation at 37 °C l-malate formation was measured by an enzymatic procedure with mMDH.

**Table 2:** Hydroxaminolysis of succinamate and l-2-Hydroxyglutaramate (l-2-HGM) catalyzed by rat liver cytosol in the absence or presence of an inhibitor ^a^.

Addition	Hydroxamate Formation nmol/min/mg
Succinamate (50 mM)	17 ± 2
Succinamate + glycylglycine (30 mM)	8.9 ± 0.8 ^b^
Succinamate + l-2-HSM (50 mM)	12 ± 1 ^c^
l-2-HGM	26 ± 1
l-2-HGM + glycylglycine (30 mM)	3.6 ± 0.2 ^d^
l-2-HGM + l-2-HSM (25 mM)	18 ± 1 ^d^

^a^ The reaction mixture (50 μL) contained 25 mM l-2-HGM (or 50 mM succinamate), 100 mM potassium phosphate (pH = 7.4), 1 mM DTT, 100 mM hydroxylamine, 5 μL of rat liver cytosol (39 mg protein/mL) and, where indicated, glycylglycine or l-2-HSM. After incubation at 37 °C for 1 hour, hydroxamate formation was measured as described in the text. n = 5; ^b^ Significantly different from the control (succinamate only) with *p* < 0.00001; ^c^ significantly different from the control (succinamate only) with *p* < 0.0005; ^d^ significantly different from the control (l-2-HGM only) with *p* < 0.00001.

**Table 3:** Hydrolysis of 2-oxoglutaramate (2-OGM) to 2-oxoglutarate (2-OG) catalyzed by rat liver cytosol in the absence or presence of an inhibitor ^a^.

Addition	2-OG Formation nmol/min/mg
2-OGM (3 mM)	13 ± 1
2-OGM + glycylglycine (30 mM)	7.9 ± 0.7 ^b^
2-OGM + l-2-HSM (25 mM)	4.5 ± 0.5 ^b^
2-OGM + succinamate (50 mM)	2.9 ± 0.2 ^c^

^a^ The reaction mixture (50 μL) contained 3 mM 2-OGM, 1 mM DTT, 100 mM tris (pH = 8.0) and 5 μL rat liver cytosol. After incubation at 37 °C for 15 minutes, 2-OG formation was measured as indicated in the text. In some cases, l-2-HSM, glycylglycine or succinamate was included in the reaction mixture. N = 5 or 6. ^b^ Significantly different from the control (2-OGM only) with *p* < 0.00001; ^c^ significantly different from the control (succinamate only) with *p* < 0.00001.

## Supplementary Materials

The following are available online at www.mdpi.com/2079-7737/6/2/24/s1, Figure S1A: ^1^H NMR spectrum of l-2-hydroxyglutaramic acid (l-2-HGM) produced from l-glutamine. The stereochemistry is assumed to be in accordance with the literature; Figure S1B: ^13^C-NMR spectrum of l-2-hydroxyglutaramic acid (l-2-HGM) produced from l-glutamine. The stereochemistry is assumed to be in accordance with the literature; Figure S2A: ^1^H NMR spectrum of l-2-hydroxysuccinamic acid (l-2-HSM) produced from l-asparagine. The stereochemistry is assumed to be in accordance with the literature; Figure S2B: ^3^C-NMR spectrum of l-2-hydroxysuccinamic acid (l-2-HSM) produced from l-asparagine. The stereochemistry is assumed to be in accordance with the literature; Figure S3: Time course for ring openings of (*S*)-(+)-5-oxo-tetrahydrofurancarboxylic and (*R*)-(-)-5-oxo-tetrahydrofurancarboxylic acids. Stock solutions of approximately 100 mM (*S*)-(+)-5-oxo-tetrahydrofurancarboxylic acid and (*R*)-(-)-5-oxo-tetrahydrofurancarboxylic acid were prepared in water (pH 1.9). The solutions were portioned into 0.2 mL samples and incubated at 80 °C for varying times. At the indicated times the sample were removed and placed on ice. Fifteen μL aliquots of the samples were then combined with 235 μL water and 750 μL 5 mM FeCl_3_. The resulting change in absorbance was measured at 428 nm, from which the concentration of ring-opened products l-2-HG (panel A) and d-2-HG (panel B) was calculated. Shown are the mean and standard deviation of between 4 and 8 measurements for each time point, Figure S4: Spectra for ferric ion complexed with l-lactate and l-2-hydroxyglutarate (l-2-HG). The spectra are of either 2.5 μmol l-lactate (lithium salt) or 2.5 μmol l-2-HG combined with 3.5 μmol FeCl_3_ in 1 mL water. The blank is 3.5 μmol of FeCl_3_ in water. l-2-HG was prepared by heating 100 mM (*S*)-(+)-5-oxo-tetrahydrofurancarboxylic acid for 24 hours at 80 °C; Figure S5: Standard curve for lactate-ferric ion complex. Dilutions of lithium lactate were prepared in water and then combined in 250 μL lots with 750 μL FeCl_3_. Shown are the mean and standard deviation of between 6 and 11 measurements.
