# Supplementary material for: The Enzymology of 2-Hydroxyglutarate, 2-Hydroxyglutaramate and 2-Hydroxysuccinamate and Their Relationship to Oncometabolites
Source: Biology (Basel). 2017 Mar 30;6(2):24. doi: 10.3390/biology6020024 (PMC5485471; doi:10.3390/biology6020024)
Supplement: Supplementary file 1 [file biology-06-00024-s001.pdf]

Supplementary Materials: The Enzymology of 2-Hydroxyglutarate, 2-Hydroxyglutaramate and 2-Hydroxysuccinamate and Their Relationship to Oncometabolites

Vivek A. Hariharan, Travis T. Denton, Sarah Paraszczak, Kyle McEvoy, Thomas M. Jeitner, Boris F. Krasnikov and Arthur J. L. Cooper

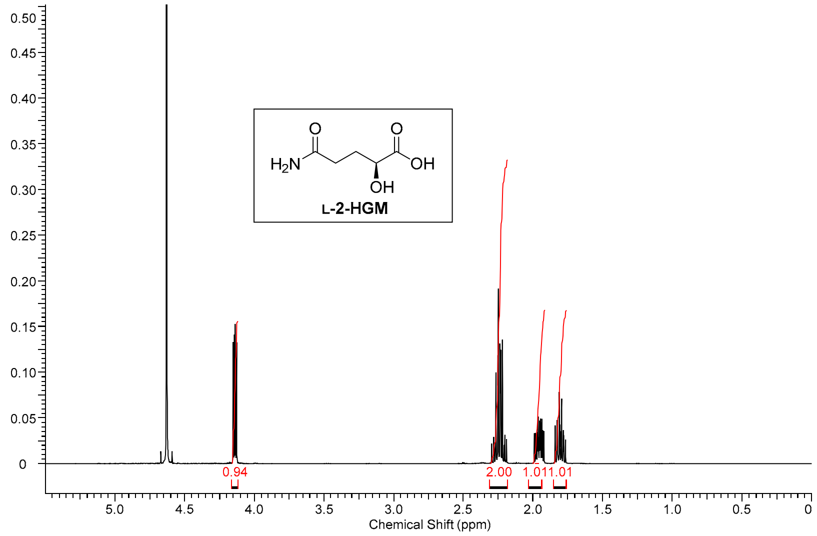

Figure S1A. ^{1}H NMR spectrum of L-2-hydroxyglutaramic acid (L-2-HGM) produced from L-glutamine. The stereochemistry is assumed to be in accordance with the literature.

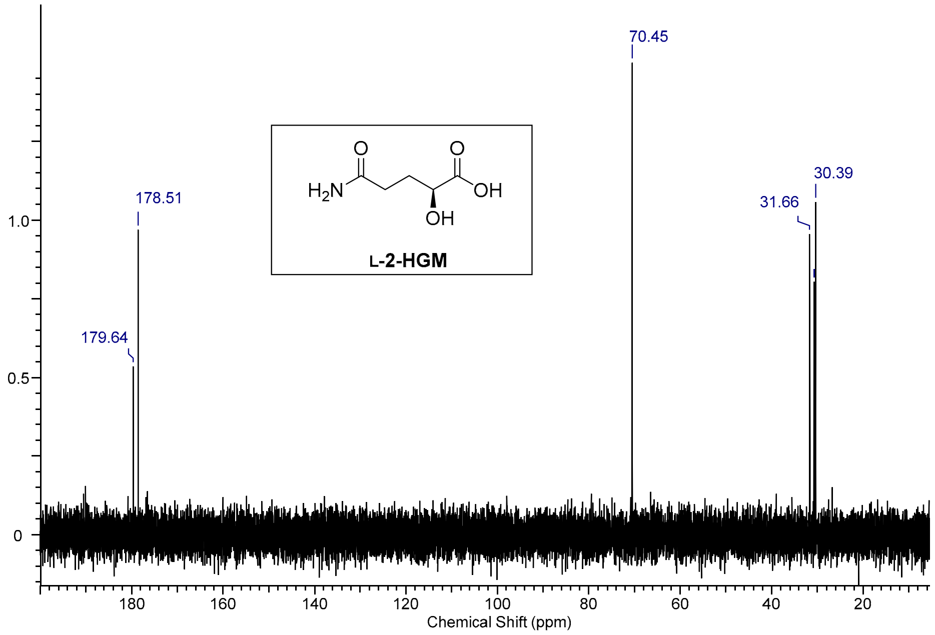

Figure S1B. ^{13}C-NMR spectrum of L-2-hydroxyglutaramic acid (L-2-HGM) produced from L-glutamine. The stereochemistry is assumed to be in accordance with the literature.

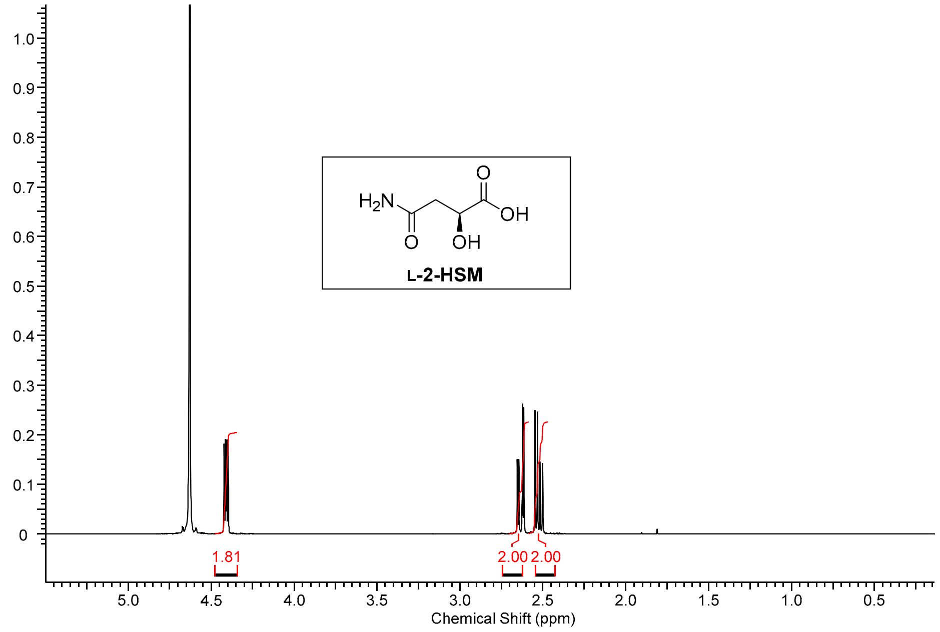

Figure S2A. ^{1}H NMR spectrum of L-2-hydroxysuccinamic acid (L-2-HSM) produced from L-asparagine. The stereochemistry is assumed to be in accordance with the literature.

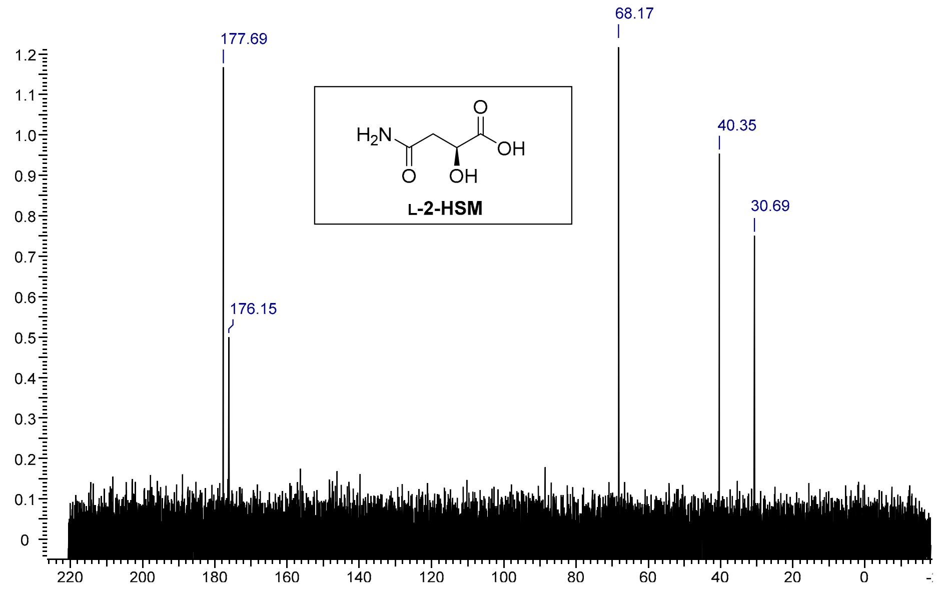

Figure S2B. ^{13}C-NMR spectrum of L-2-hydroxysuccinamic acid (L-2-HSM) produced from L-asparagine. The stereochemistry is assumed to be in accordance with the literature.

Preparation of the L- and D-enantiomers of 2-hydroxyglutaric acid

The disodium salt of L-2-hydroxyglutaric acid (Sigma Aldrich Chemical Company, St. Louis, MO, USA) and sodium salt of D-hydroxyglutaric acid (Cayman Chemical Company, Ann Arbor, MI, USA) have recently become available commercially. However, they are very expensive. L- and D-2-hydroxyglutaric acids were therefore prepared less expensively from the corresponding lactones [(*S*)-(+)-5-oxo-tetrahydrofurancarboxylic acid and (*R*)-(-)-5-oxo-tetrahydrofurancarboxylic acid, respectively]. Stock solutions (100 mM) of the lactones (pH 1.9) were heated at 80 °C until opening of the ring was complete (**Figure S3**). The method for determining extent of ring opening is as follows: At intervals, 15 µL aliquots were withdrawn and assayed for product formation by a colorimetric procedure. The assay procedure is based on ring opening to yield a 2-hydroxy

carboxylic acid. It has been known for over 120 years that lactic acid (a prototypical 2-hydroxy acid) turns a dilute solution of ferric chloride to a deep yellow color [Hewes H.F. The chemical analysis of the gastric content. Boston Medical and Surgical Journal (1897) 137, 565-9.] The ferric complex with lactic acid exhibits a maximum absorbance at 428 nm (**Figure S4**). It was found that the lactones exhibit negligible absorbance at 428 nm in the presence of dilute ferric chloride. However, when ring opening is complete the absorbance of the ferric complex of the resulting L-2-hydroxyglutaric acid is identical within experimental error to that exhibited by L-lactic acid (**Figure S4**). A standard curve of absorbance at 428 nm versus concentration of L-lactic acid is shown in **Figure S5**. This curve was used to estimate the rate of conversion of the lactones to the corresponding 2-hydroxy acids. It was found that ring opening of a 100 mM solution of both [(*S*)-(+)-5-oxo-tetrahydrofurancarboxylic acid and (*R*)-(-)-5-oxo-tetrahydrofurancarboxylic acid enantiomers to the corresponding 2-hydroxy acids is complete in about 12 hours (**Figure S3**).

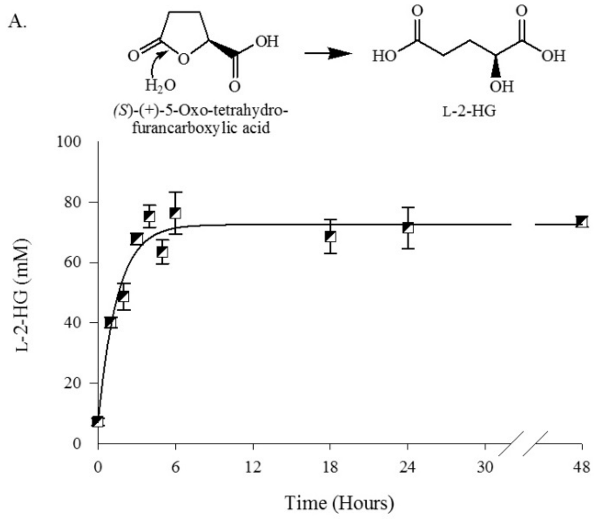

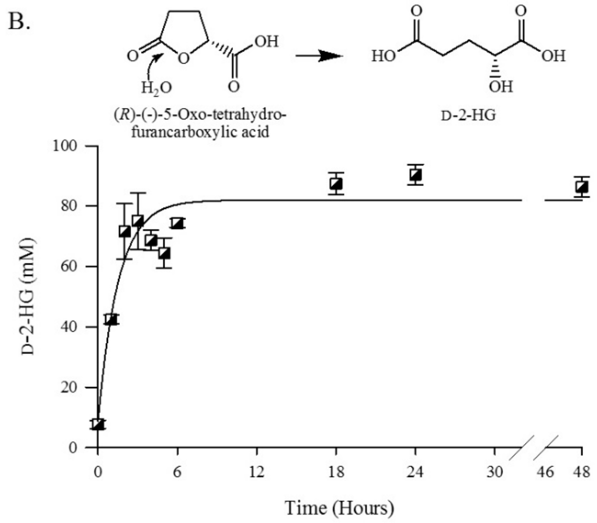

Figure S3. Time course for ring openings of (*S*)-(+)-5-oxo-tetrahydrofurancarboxylic and (*R*)-(-)-5-oxo-tetrahydrofurancarboxylic acids. Stock solutions of approximately 100 mM (*S*)-(+)-5-oxo-tetrahydrofurancarboxylic acid and (*R*)-(-)-5-oxo-tetrahydrofurancarboxylic acid were prepared in water (pH 1.9). The solutions were portioned into 0.2 mL samples and incubated at 80 °C for varying times. At the indicated times the sample were removed and placed on ice. Fifteen µL aliquots of the samples were then combined with 235 µL water and 750 µL 5 mM $FeCl_3$. The resulting change in absorbance was measured at 428 nm, from which the concentration of ring-opened products L-2-HG (panel A) and D-2-HG (panel B) was calculated. Shown are the mean and standard deviation of between 4 and 8 measurements for each time point.

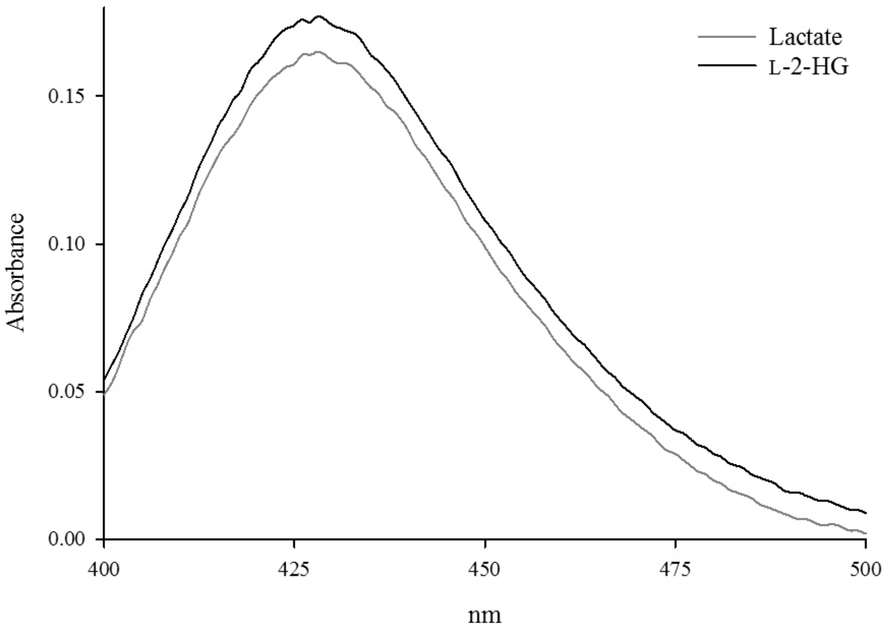

Figure S4. Spectra for ferric ion complexed with L-lactate and L-2-hydroxyglutarate (L-2-HG). The spectra are of either 2.5 µmol L-lactate (lithium salt) or 2.5 µmol L-2-HG combined with 3.5 µmol $FeCl_3$ in 1 mL water. The blank is 3.5 µmol of $FeCl_3$ in water. L-2-HG was prepared by heating 100 mM (*S*)-(+)-5-oxo-tetrahydrofurancarboxylic acid for 24 hours at 80 °C.

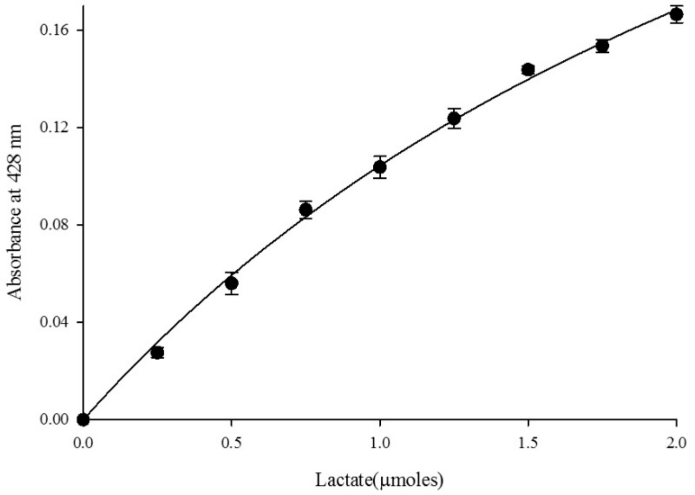

Figure S5. Standard curve for L-lactate-ferric ion complex. Dilutions of lithium lactate were prepared in water and then combined in 250 µL lots with 750 µL $FeCl_3$ solution. Shown are the mean and standard deviation of between 6 and 11 measurements.
